# Psychosocial distress and psychological adjustment in patients with ocular loss: a framework analysis

**DOI:** 10.1186/s12903-022-02597-1

**Published:** 2022-11-24

**Authors:** Natdhanai Chotprasert, Binit Shrestha, Patcharanin Thanasapburachot, Rattakan Kanpiputana, Kawin Sipiyaruk

**Affiliations:** 1 Maxillofacial Prosthetic Clinic, Department of Prosthodontics, Faculty of Dentistry, Mahidol University, Bangkok, Thailand; 2 Research Office, Faculty of Dentistry, Mahidol University, Bangkok, Thailand; 3 Department of Orthodontics, Faculty of Dentistry, Mahidol University, Bangkok, Thailand

**Keywords:** Dental education, Facial disfigurement, Maxillofacial prosthetics, Outcome-based education, Patient-centered care psychological adjustment

## Abstract

**Background:**

Patients with ocular loss tend to have physical and psychosocial difficulties. Maxillofacial prosthetic specialists involved in the treatment should be trained with competence to manage psychological complications. However, due to the multifactorial origin of the psychosocial distress, designing such training activities can be challenging. This research aimed to construct a conceptual framework illustrating the effects of ocular loss on patients’ physical and psychosocial well-being and their coping strategies, to propose the learning content of training sessions.

**Methods:**

A semi-structured interview and a topic guide were employed to explore the perceptions from patients who were receiving their first custom ocular prosthesis, fabricated by maxillofacial prosthodontists. The participants were selected using a purposeful sampling up until data saturation. The data were analyzed using framework analysis.

**Results:**

Twelve patients participated in this research. Four main themes emerged from the data analysis: (1) Impact of ocular loss, (2) Factors influencing psychosocial distress, (3) Psychological adjustment, and (4) Expected treatment outcomes. Each theme appeared to have influence on the other, rather than presenting itself independently. Although patients with ocular loss experienced both physical and psychosocial difficulties, psychological distress was greatly influenced by self-perceived disfigurement, etiology, and social status. Therefore, they needed to develop their coping strategies including rehabilitation with ocular prosthesis.

**Conclusion:**

Various forms of psychological adjustments were necessary in these patients with ocular loss to resume their daily lives. The specialist involved in the treatment should also partake in patients’ psychological adjustment and should be competent in psychological management skills, such as supporting patients to meet their expectations.

## Introduction

Out of all the sensory organs, eyes are the most crucial to navigate and learn about the world. Humans also use eyes to communicate or express their feelings, such as happiness, sadness, or anger. In addition, they are perceived as an esthetic component of facial appearance. Characteristics of the eye-size or inter-eye distance, have influence on perceived facial appearance [1, 2]. Furthermore, they are likely to influence not only physical but also psychosocial aspects.

A number of patients have been reported with a loss of one or both eyes due to trauma, end-stage eye diseases, tumors, or congenital malformations [3]. Its loss can negatively affect physical appearance, known as ‘facial disfigurement’, leading to psychological impairment. Visible differences in physical appearance from the norm or ideal characteristics can result in psychological problems, such as embarrassment, anxiety, and depression [4]. Patients with ocular disfigurement can experience psychological problems, and, therefore, need ways to relieve their mental distress, known as ‘psychological adjustment’.

A sequence of psychological adjustments can be initiated in these patients by wearing an ocular prosthesis. According to Goiato et al. [5], patients reported they felt less depressed following ocular prosthetic rehabilitation; however, the satisfaction level varied among individuals. In other words, the extent of psychological adjustment can be governed by numerous factors. Interestingly, psychological adjustment could not be fully predicted by etiology and severity of disfigurement [4]. Good predictors of adjustment are psychosocial factors, such as perceptions of social support and acceptance as well as self-perceived appearance [4, 6, 7]. Therefore, specialists involved in ocular rehabilitation, such as ocularists, prosthetists, or maxillofacial prosthodontists, are required to have competence in the management of both physical and psychological difficulties in these patients.

Based on a review of literature, there appeared to be a scarcity of research regarding the psychological well-being of patients with ocular loss, especially in terms of disfigured appearance. Although members of the treatment team, including maxillofacial prosthodontists should be competent in dealing with psychological distress, there are several challenges in designing training activities for either healthcare undergraduates or postgraduates. Hence, this research aimed to qualitatively explore physical and psychosocial challenges in patients with ocular loss as well as their coping strategies, in order to construct a conceptual framework demonstrating a psychological adjustment model to propose the expected learning outcomes and required content of training activities.

## Materials and methods

### Research design

This study employed a qualitative paradigm, using semi-structured interviews with a topic guide. This technique allows researchers to collect relevant data and at the same time investigate the breadth of new concepts [8]. The topic guide for this study was constructed based on previous literature with modifications [5, 9–12]. For instance, the questions regarding social avoidance were taken from the Social Avoidance and Distress Scale, which is a set of questions used to measures social anxiety including distress and avoidance in social situations [9]. The first part of the topic guide aimed to explore the patient’s profile and history of ocular loss. The subsequent parts were constructed to investigate both physical and psychological aspects of participants, covering their difficulties with daily tasks, self-perceived appearance, social avoidance, anxiety, and depression. Participants were also asked about their coping strategies with their challenges and difficulties. Finally, their expectations towards ocular prosthesis were explored.

### Sample selection and recruitment process

Participants in this study were adult patients with monocular eye loss (age: 18 or above) who were undergoing their first ocular rehabilitation with a custom ocular prosthesis, fabricated by maxillofacial prosthodontists at the Maxillofacial Prosthetic Clinic, Mahidol University. The exclusion criteria were individuals who had previously worn an ocular prosthesis and had eye loss for a period shorter than a year. They were selected for a semi-structured interview using purposeful sampling by considering patient information up until data saturation, where the medical record was screened by a member of the research team who was expert in maxillofacial prosthetics (NC). This technique enabled the researchers to select information-rich samples [13, 14]. For instance, the sample selection had an emphasis on patients who requested an ocular prosthetic rehabilitation, rather than being referred from other healthcare professionals. In addition, with over a year of experience on an eye loss, they would be able to provide their in-depth responses towards probing questions during an interview. The sample selection also considered age, sex, marital status, and occupation, in order to address the variation of the research participants.

Following the sample selection, the lists of proposed participants were created and coded, where support staff was requested to randomly select and indirectly recruit patients who were awaiting ocular prosthetic rehabilitation, leading to the minimum of selection bias. This procedure also ensured that the participants were keen to participate in an interview, which could reduce response bias, as their decision to participated in this research was completely voluntary.

### Data collection process

The interview took approximately 45 min in an enclosed private space at the Maxillofacial Prosthetic Clinic. Written informed consent was obtained from all participants prior to the interview. All interview sessions were conducted by a researcher who was well trained in conducting social science research (KS) and had no conflict of interest with patients in the clinic. This would minimize the risk of response bias. All responses during interviews were recorded using a digital voice recorder in agreement with the research participants, and recordings were transcribed using a verbatim technique to preserve actual words, intonation, and rhythm. Any identifiable data were anonymized to maintain confidentiality of research participants. The interview was conducted in Thai language. However, the transcripts were translated to English and, then, translated back to Thai. After that, both Thai versions were checked to examine whether any revisions of the translation were required, to enhance the validity of the translation process.

### Data analysis

The data were analyzed using framework analysis, which was involved with the rigorous and transparent process of data charting and sorting to manage, compare, and interpret data of emerging themes [15]. This approach was selected, as its transparency allowed two researchers (NC and KS) to work collaboratively during the process of data analysis, in order to assure the internal validity and intercoder reliability of this research. The initial themes for the analysis were derived from the topic guide, developed from a review of literature. All emerging themes and subthemes as well as their relationships were based on an agreement of the two researchers, including the decision on data saturation, where there was no new substantive information acquired from research participants. The data analysis was performed using NVivo (QSR International), which is a computer software supporting researchers to organize and analyze qualitative data.

### Ethical considerations

In order to minimize the pressure on the decision of research participation, the proposed participants were indirectly recruited by support staff from a queue of patients awaiting ocular prosthetic rehabilitation treatment at the Maxillofacial Prosthetics Unit, Faculty of Dentistry, Mahidol University, Bangkok, Thailand. Any of their decisions would not affect the treatment. Informed consent was obtained from all participants prior to the interviews. This research protocol was approved by the Faculty of Dentistry/Faculty of Pharmacy, Mahidol University Institutional Review Board, reference number: MU-DT/PY-IRB 2018/DT018. All methods were performed in accordance with the relevant guidelines and regulations.

## Results

There were 12 patients with monocular eye loss who participated in this study. The characteristics of the participants was presented in Table 1. Following the qualitative data analysis, four main themes emerged, which were (1) Impact of ocular loss, (2) Factors influencing psychosocial distress, (3) Psychological adjustment, and (4) Expected treatment outcomes.

**Table 1:** Sample characteristics

Participants	Age	Marital status	Level of education	Occupation	Etiology of eye loss	Duration of eye loss (years)
Female 1	22	Single	Studying bachelor’s degree	Student	Accident	7
Female 2	47	Married	Bachelor’s degree	Housewife	Accident	6
Female 3	68	Married	High school	Retired	Infection	20
Female 4	66	Married	High school	Retired	Accident	40
Female 5	20	Single	Studying bachelor’s degree	Student	Tumor	5
Female 6	62	Married	Bachelor’s degree	Teacher	Tumor	20
Male 1	71	Married	Bachelor’s degree	Retired	Macular degeneration	3
Male 2	22	Single	Bachelor’s degree	Student	Accident	4
Male 3	26	Single	Bachelor’s degree	Employee	Congenital loss	26
Male 4	43	Married	Bachelor’s degree	Employee	Accident	13
Male 5	32	Married	High school	Employee	Accident	4
Male 6	61	Single	Bachelor’s degree	Retired	Accident	8

### Impact of ocular loss

#### Physical impairment

All participants seemed to experience some difficulties in their everyday lives with monocular vision. As there were limitations in the field of vision and depth perception, they reported that they struggled to perform normal activities, such as driving, playing sports, ascending or descending stairs, or even walking on a flat ground. However, they were likely to develop strategies to cope with their physical problems, such as seeking ocular rehabilitation, self-help, or peer-related support, and could resume their daily tasks afterwards.

It (eye loss) causes me to do activities much more slowly … when walking down the stairs or even on a flat ground. I have difficulties in perceiving the distance between the steps.

Female 2, 47 years old

I do not yet feel confident in driving a car on main roads. With a restricted field of vision, there are limitations when turning the car or changing lanes.

Male 3, 26 years old

#### Psychosocial distress

##### Stress

Stress appeared to be a common mental problem especially in participants with acquired ocular loss, as their routine lifestyle or career security was greatly affected by functional adequacy from visual difficulties.

It was quite stressful at that time, when I just lost my eye. With the vision loss, I had difficulties with everyday tasks.

Female 1, 22 years old

##### Anxiety

Participants tended to have anxiety and feared that their disfigurement could have negative affect on their relationship with others. They believed that their ocular loss was a reason why their job applications were rejected. In addition, they thought that they broke up with their partner probably due to their impairment or disfigurement.

It was slightly painful when my job application was rejected. I thought my eye loss could be a reason.

Male 4, 43 years old

I had fear that my eye loss would lead to the breakup with my partners. I also thought that she should not have had to spend difficult time with me.

Male 5, 32 years old

##### Depression

Participants also felt depressed with their sudden eye loss mostly from accidents or trauma with foreign objects. Some lost interest in their everyday life.

When I found out that my eye needed to be removed, I cried a lot and I did not know what I should do with my life … [crying] … I did not want to do anything even my daily tasks.

Female 5, 20 years old

##### Social avoidance

Another common psychological distress seemed to be social avoidance. Participants reported that they were worried to be in public. If it was absolutely necessary, they were likely to wear eyeglasses to hide their disfigurement. They felt uncomfortable when being stared at or asked about their disfigurement. One participant reported that she was worried others would feel embarrassed of her.

I found my disfigured appearance is different from others … I need to wear glasses all the time that I am outside. So, I feel more comfortable to stay at home, rather than going outside to do activities, to see friends, …

Male 6, 61 years old

I was afraid that my husband would feel ashamed of my disfigurement in front of his friends.

Female 4, 66 years old

### Factors influencing psychosocial distress

#### Etiology of ocular loss

The etiology of ocular loss appeared to affect the level of psychosocial distress. Participants with congenital eye loss were likely to efficiently cope with their psychological problems. As they grew up with monocular vision, they were familiar to live with only the remaining eye. Similarly, participants with gradual ocular loss had a period of time for psychological adjustment.

I have never had this eye since I was born, and I am used to it.

Male 3, 26 years old

I felt disappointed just for a while. It was a bit lucky that it was a gradual loss.

Male 1, 71 years old

Participants who experienced a sudden loss of the eye (e.g., trauma or malignancy) reported they had a difficult time to adapt themselves to resume their daily lives. Since the loss occurred rapidly, these patients struggled to overcome both physical and psychological difficulties.

When I was told that I had to remove the eye even though it was still functioning, I was not able to deal with it … I cried a lot.

Female 5, 20 years old

#### Social status

The social status of patients can also influence psychological distress. Participants who were studying or working reported that they did not feel confident of being accepted at their workplace. They also worried about their job security, which could be affected by visual impairment or facial disfigurement. Moreover, career position also related to the level of distress.

I was a deputy director, so it would be a bit strange if I had a disfigured face.

Female 6, 62 years old

If the job does not require meeting with customers or something like that, it should be fine.

Female 1, 22 years old

#### Self-perceived disfigurement

Compared to the severity of disfigured appearance, self-perceived disfigurement appeared to be an important influential factor for psychological distress. In other words, the level of psychological distress depended on how these individuals perceived their appearance. This was also relevant to how important the appearance was in their own perspective.

I now feel very disappointed with how I look (compared to others). I want to be like how I was before … [crying] … to be outside without glasses.

Female 2, 47 years old

Physical appearance plays an important role for me. I always took care of my body … to look good in public ... but now …

Female 4, 66 years old

### Psychological adjustment

Following the visual impairment and facial disfigurement, participants reported that they were able to adapt themselves to the changes, leading to improvement in psychological well-being. The key strategies reported by the participants were as follows:

#### Ocular prosthetic rehabilitation

Participants believed that ocular prosthesis would restore their facial disfigurement, supporting them to resume their daily life. They considered that the ocular prosthesis would make them feel more confident to interact with others and relieve their psychological distress.

If I have the prosthesis, I should not have a problem in making a positive first impression for my job interview.

Female 1, 22 years old

#### Self-help coping strategies

Several participants reported they had enjoyable and relaxing leisure activities, either indoors or outdoors, to relieve their emotional distress, such as stress, anxiety, and depression.

Just listening to music, doing exercise, and playing sports.

Male 2, 22 years old

#### Peer-related psychosocial support

Peer-support plays an integral role in reducing psychosocial distress. Words of encouragement from family members and friends appeared to be very supportive to the patients to cope with their mental distress. A social community or group where members could share similar interests or problems with others can also be an effective way to gain psychological support.

Immediately after the eye removal, my partner stayed with me all the time … to give me a hand when I needed help.

Male 4, 43 years old

I would say… trainings for blind people. It did not only help me perform my daily tasks, but also I could talk with others who had similar problems. Several activities were designed for impaired people.

Female 3, 68 years old

### Expected treatment outcomes

#### Expectations towards ocular prosthesis

Patients’ expectation towards an ocular prosthesis appeared to be varied, which could impact the level of psychological adjustment. Higher expectations might negatively affect satisfaction towards an ocular prosthesis, if it could not be achieved from the treatment.

I just expected it [an ocular prosthesis] to look more natural than this one [an ocular conformer].

Male 5, 32 years old

I would like the prosthetic eye to have natural movements, similar to the other side.

Female 5, 20 years old

#### Expectations towards dental professionals

Participants suggested that they would appreciate if the specialist involved in the treatment, i.e. maxillofacial prosthodontist, provided them with psychological support. Not only physical recommendations but also psychological advice could be encouraging to them.

Firstly, I would expect my doctor to understand my situation, and if they could provide words of encouragement, it would be very supportive.

Male 4, 43 years old

## Conceptual framework of psychological adjustment

**Fig. 1:**
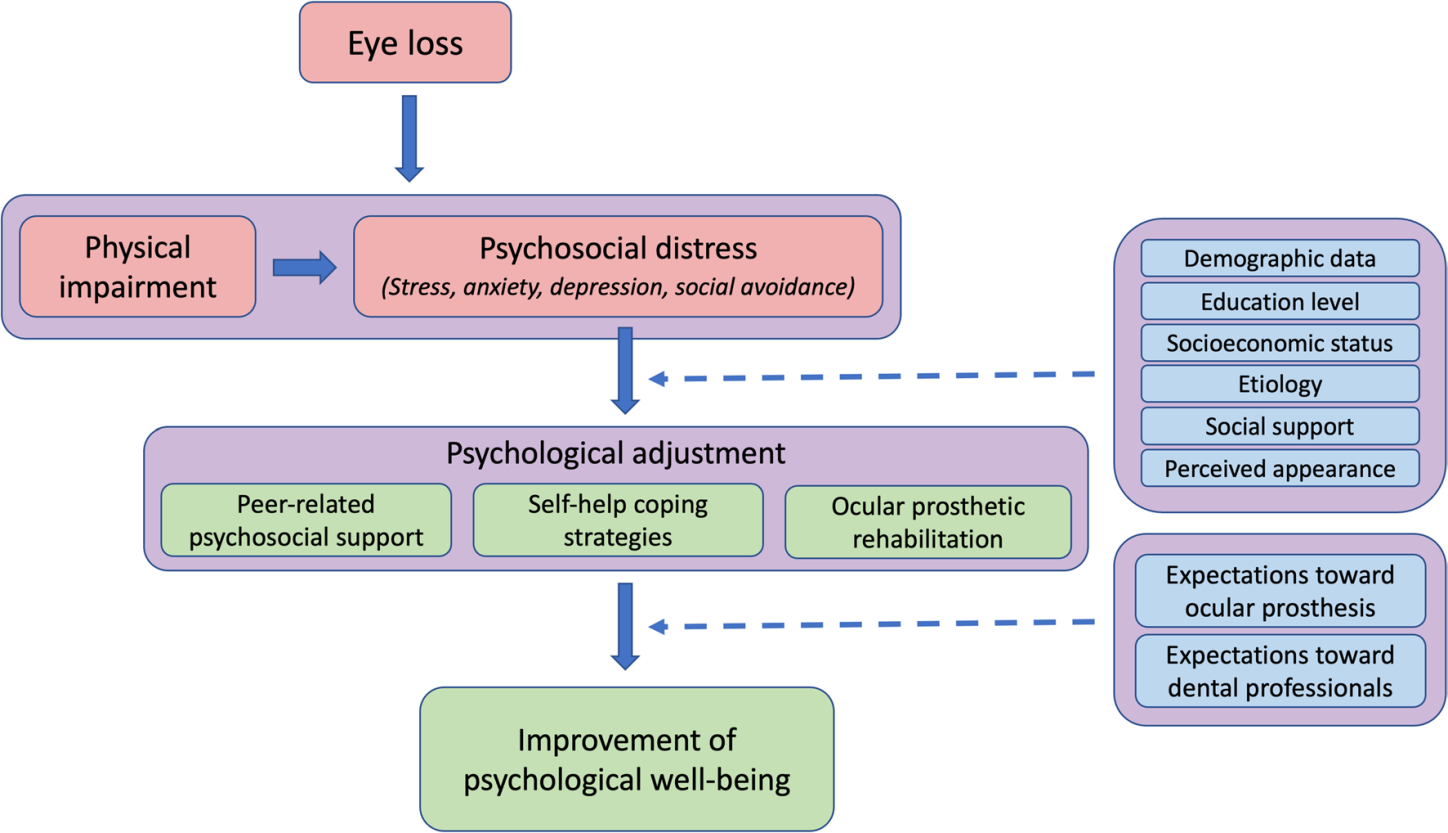
Conceptual framework of psychological adjustment retrieved from the study

Based on the qualitative findings, a conceptual framework was developed in which psychological adjustment was conceptualized as a strategy to address physical and psychosocial challenges in patients with ocular loss, as presented in Fig. 1. The framework also demonstrates the relationships of the identified themes, as well as the influential factors which could impact the improvement of psychological well-being.

## Discussion

Ocular loss was found to have negative impacts on the physical and psychological well-being of the patients, as evident from the results of this research. Although some patients could still see with the remaining eye, monocular vision led to physical impairments. Their motor functions were likely to decrease with monocular vision, leading to restrictions in performing daily activities [16, 17]. In addition, there was a greater risk of depression in people with visual impairment [18]. These factors could, in turn, negatively affect their quality of life.

It was observed that both physical impairment and disfigured appearance could adversely impact psychological well-being. Our findings were consistent with previous studies on this field [4, 19–21], which have noted that psychological well-being was not only affected by the severity of disfigurement but also other influential factors including demographic data (age and sex), education level, socioeconomic status, marital status, etiology, self-perceived appearance, as well as perceptions of social support and acceptance. Females tended to suffer from higher degree of depression, requiring more social support than males [19]. As facial disfigurement can lead to psychological distress, patients with ocular loss are required to develop coping strategies to promote their well-being.

In addition to peer-related psychosocial support and self-help coping strategies, the participants expected that the ocular prosthesis would restore the esthetic balance by hiding their disfigurement or visible differences. Ocular prosthesis has been found to have a positive impact on psychosocial health [22]. When patients could balance or adapt their psychological needs and resume their daily activities, it meant that their needs were adjusted, following the concept of psychological adjustment.

Patients’ expectations towards ocular prosthesis can be varied, which could impact the level of satisfaction. Even though a prosthesis is successfully delivered, psychosocial distress may continue to remain in patients with eye loss [23]. Satisfaction with facial appearance was found to be correlated with self-esteem and social functioning, rather than objective improvement of the disfigurement [24]. Some of the patient’s expectations can be beyond the limit of the prosthesis, therefore, the personnel involved in the ocular rehabilitation (i.e. ocularist, prosthetist, or maxillofacial prosthodontist) should thoughtfully educate the patients to rid of any misconceptions that the patient might have prior to initiating the treatment. Nonetheless, ocular rehabilitation is the initial step towards recovery of patients’ overall well-being.

The qualitative findings of this research provided in-depth knowledge regarding the psychological dimensions of patients with ocular loss and enabled the development of a conceptual framework illustrating the relationships between identified variables (Fig. 1), which was the key objective of this research. This model could enhance our understanding in psychosocial distress and psychological adjustment, which could be used as a guideline or regulations in delivering ocular prosthetic rehabilitation. For the betterment of patient care, maxillofacial prosthetic specialists should evaluate physical and psychosocial challenges of patients and identify any other factors which may have direct or indirect influence on the treatment outcomes. It can also be a useful framework for academic staff and educators to identify expected learning outcomes and to design training activities, enabling the maxillofacial prosthetic specialists to be competent in providing psychological support and management to patients with disfigurement. For instance, they should be able to not only assess physical severity but also to evaluate how their patients could manage psychological distress.

One of the strengths of this research was our design to recruit participants who had experienced ocular loss for at least a year or longer, which enabled us to understand the patients’ perceptions towards ocular loss and treatment expectations. In addition, this research selected participants with a wide variety of characteristics to maximize data extension. Therefore, our findings could be adapted to other environments with similar context. However, as visual acuity of the remaining eye was not investigated in this study, further research should explore whether or not it could have impact on psychosocial distress and psychological adjustment. Although the conceptual framework constructed from this qualitative study could be used as a guideline in providing ocular prosthetic rehabilitation, further quantitative research should be required to develop a statistical model to demonstrate influential factors of psychosocial distress and their adjustment, as well as to enhance the generalizability.

## Conclusions

Ocular loss had negative impacts on the physical and psychological well-being of the patients. Their psychosocial distress could not be evaluated by their disfigurement alone, but the consideration of other influential factors, including socioeconomic status, social support, and self-perceived appearance, should be required. While psychological adjustment including rehabilitation with ocular prosthetics was important to the patients to resume their daily lives, their expectations towards the treatment outcome could be different. Consequently, during training activities these issues should be addressed such that the specialist involved in the treatment can proficiently provide care and support to these patients to meet the expected treatment outcomes.

## Data Availability

The datasets generated and/or analyzed during the current study are not publicly available due to confidentiality reasons and ethical considerations but are available from the corresponding author on reasonable request.
